# Hospital and Health News

**Published:** 1924-09

**Authors:** 


### HOSPITAL AND HEALTH NEWS.

### A Centenarian Coroner.

Dr. Edwin J. Slade-King, coroner for the County of Devon and Medical Officer of Health for Ilfracombe, will soon be 100 years old.

### Food Adulteration.

The Medical Officer of Health for Middlesex states that out of 849 samples inspected under the Food and Drugs Act during 1923, 106 were found to be adulterated.

### The Royal Gwent Hospital.

On the August Bank Holiday, a favourite day for ceremonies at the Royal Gwent Hospital, Newport, Lord Tredegar opened the new out-patient department.

### Hospital Saturday and Sunday.

On September 6 next Hospital Saturday will come round for the fifty-first time. Last year the sum collected amounted to £100,889. The total collection on Hospital Sunday this year amounted to £84,000, or practically the same as that of 1923.

### Help from Spain.

One hundred pounds has been sent for the foundation of the Ross Institute and Hospital for Tropical Diseases, 33 Harley Street by the Municipal Council of Gibraltar.

### Orchid Juice for Consumption.

At the recent Congress at Liège, Dr. Barbier, a scientist, claimed that tuberculosis could be successfully treated by giving the patient juice extracted from the corolla of the orchid.

### More Medical Books for Wigan.

A distinguished medical practitioner, who wishes to remain anonymous, has endowed Wigan Public Library with £400 worth of 5 per cent. stock, the income from which is to be utilised for the purchase of important medical works for the reference department.

### A Ward for Snorers.

At the Nuneaton Poor Law Institution there is a special ward for male inmates who snore. A Yorkshire newspaper, describing it, says: "The noise that comes from it is like the roaring of many lions . . . but the other inmates have peace."

### Death of Mr. J. H. Read.

The recent death of Mr. J. H. Read, the permanent private secretary to the Lord Mayors of Bristol, removes the initiator of the Lord Mayor's Hospital Fund which was established there in 1903. Mr. Reed was a retired schoolmaster with a keen interest in botany and local history and traditions.

### A Harrogate Centenary.

The Harrogate Royal Hospital, one of the three institutions in England devoted to the treatment and investigation of rheumatism, celebrates its centenary on September 3. A sum of £12,000 is required for further extensions, and purses are being collected in towns from which patients have lately been treated.

### Strengthening London's Ambulance Service.

London's ambulance service, according to the London County Council report for 1923, is being strengthened by the erection of subsidiary stations at Battersea, Paddington, Highbury and Woolwich, which will raise the number from seven to eleven. The summer months are always the busiest time.

### Bethnal Green Hospital and an Inquiry.

The Bethnal Green Borough Council has passed unanimously a resolution criticising the Guardians for their administration of Bethnal Green Hospital and, as the local sanitary authority, asking what steps the Guardians are taking to remedy the inadequacy of staff, equipment, and buildings. Scathing comments were made, and a public inquiry is expected.

### "The Cripples Journal."

The increased importance of orthopædic work and the interest which it is beginning to occupy in the public mind is shown by the issue of a new quarterly magazine entitled "The Cripples Journal." Clinics for the care of crippled children are springing up all over the country, and the journal will be a valuable link between the various workers in these centres and will also advise on the establishment and maintenance of new clinics. Sir Robert Jones, who has done so splendid a work in this direction, contributes an article on its future.

### Site for Royal Ear Hospital.

A site on the corner of Huntley Street and Pancras Street has been purchased for the rebuilding of the Royal Ear Hospital—conveniently close to University College Hospital, of which it is a branch, and the new Rockefeller buildings. A thoroughly up-to-date ear, nose and throat hospital is proposed with facilities for research.

### Resignation of Mr. C. S. Risbee.

Mr. Carrington S. Risbee has retired from the secretaryship of the Northampton General Hospital, which he has held for 27 years. When he joined the staff in 1897 the institution was still called the Infirmary; four years later a scheme was determined which added two new wings containing 156 beds at a final cost of £36,000. The old building was converted at the same time into the administration block and Nurses' Home. The reconstructed hospital was reopened formally in 1904. There have been several additions since then, the latest of which is a new ophthalmic department for out-patients.

### Instruction in Infants' Diseases.

A course in infants' diseases is to be held from September 15 to 27 at the Infants' Hospital, Vincent Square, Westminster. Besides lectures by Dr. Eric Pritchard, Dr. Donald Paterson, Dr. Norah Schuster, Dr. Helen Mackay and other authorities on mother and child welfare, there will be a number of demonstrations, consultations and occasional visits to other hospitals. The fee for the course will be £3 3s. Tea will be provided at a small charge each day. Qualified practitioners wishing to attend the course (the number will be limited to 15) should apply to the Secretary to the Fellowship, 1 Wimpole Street, W. 1.

### Two Hospital Pamphlets.

King Edward's Hospital Fund issues two little pamphlets, one on "The London Voluntary Hospitals and the Overseas Dominions," signed by distinguished medical men, and "calling the attention of those from Britain over the seas to the special claims which this work has upon their interest." The other, entitled "Answers to Correspondents," gives details of the financial position of the London Voluntary Hospitals. To this latter Mr. Pett Ridge contributes a preface, and the pamphlet is of particular value since it shows the situation at a glance.

### Sanitary Inspectors' Association.

The annual conference of the Sanitary Inspectors' Association, to be held at The Rotunda, Whitley Bay, from September 2 to September 5, will be provided over by Sir William Collins, who will deliver his address on the morning of September 3 following the official reception. In the afternoon Mr. Haygarth Brown, of the Ministry of Agriculture, will speak on "The Proposed Amendment of the Law as to Fertilisers and Feeding Stuffs," and in the evening there will be a reception of members and delegates. Other subjects for discussion include "Humane Slaughter of Animals," "Food Orders in Relation to Milk" and Housing conditions.

### London School of Tropical Medicine.

An article on the future of this school appeared in our August issue. The Division of Tropical Medicine and Hygiene has been taken over by the new body as from August 1. The Director of the School is Dr. Andrew Balfour, C B., C.M.G. The ordinary course extends over a period of twelve weeks, and is so arranged as to equip men to sit for the diplomas in Tropical Medicine of Cambridge and London, and for the M.D. in Tropical Medicine of London University. Three courses are given each year beginning September, January and April. Special courses are held in protozoology, entomology and helminthology, for those who have previously taken the ordinary curriculum. The school is housed in the building of the Hospital for Tropical Diseases, Endsleigh Gardens, Euston Road, N.W.

### A New Departure in Nursing.

The Nightingale Fund Committee established (as an experiment) a Traveller Fellowship, the holder of which is to visit the United States and Canada to examine and enquire into nursing, especially in relation to public health, and to report. Miss Olive Baggallay has been appointed to this position. Trained in the Nightingale Training School, St. Thomas's Hospital, she gained a Nightingale Scholarship for post-graduate work at King's College for Women. Experienced in practical nursing, both in hospital and on the district, she later devoted herself to the public health side, and is now working under the Battersea Borough Council.—Miss Ruth Darbyshire, R.R.C., matron of University College Hospital, has been invited by the Rockefeller Foundation to visit the hospitals in America.

### The Plaistow Charity.

The Maternity Charity of Plaistow exists for providing nurses to nurse the poor in their own homes in maternity and other cases. The women who avail themselves of the hospital and its co-related activities are the wives of men engaged in dock and riverside work, and they endeavour to pay a certain amount towards the cost of their maintenance. The Maternity Charity and District Nurses' Home, Howards Road, stands on one large piece of ground in Plaistow, where the nurses' home, the maternity hospital, and Chesterton House (maternity and child-welfare centre) are near each other, and there is a pleasant garden for the convalescent. The hospital a[illegible]d its midwives and nurses are held in honour and esteem, bu[illegible] their popularity has one drawback—there is a £[illegible],000 debt hanging over the hospital and hampering its work Gifts of 500*l.* entitle the donor to name a bed; of £1,000 to have a tablet placed in the hospital.

